# Transcriptomics and Genomics Analysis Uncover the Differentially Expressed Chlorophyll and Carotenoid-Related Genes in Celery

**DOI:** 10.3390/ijms23168986

**Published:** 2022-08-12

**Authors:** Xiaoming Song, Nan Li, Yingchao Zhang, Yi Liang, Rong Zhou, Tong Yu, Shaoqin Shen, Shuyan Feng, Yu Zhang, Xiuqing Li, Hao Lin, Xiyin Wang

**Affiliations:** 1 Center for Informational Biology, School of Life Science and Technology, University of Electronic Science and Technology of China, Chengdu 610054, China; 2 Center for Genomics and Bio-Computing, School of Life Sciences, North China University of Science and Technology, Tangshan 063210, China; 3 Beijing Vegetable Research Center, Beijing Academy of Agriculture and Forestry Sciences, Key Laboratory of Biology and Genetic Improvement of Horticultural Crops (North China), Beijing 100097, China; 4 Department of Food Science, Aarhus University, 8200 Aarhus, Denmark; 5 Fredericton Research and Development Centre, Agriculture and Agri-Food Canada, Fredericton, NB E3B 4Z7, Canada

**Keywords:** celery, chlorophyll, carotenoid, gene duplication, expression pattern, regulatory network

## Abstract

Celery (*Apium graveolens* L.), a plant from Apiaceae, is one of the most important vegetables and is grown worldwide. Carotenoids can capture light energy and transfer it to chlorophyll, which plays a central role in photosynthesis. Here, by performing transcriptomics and genomics analysis, we identified and conducted a comprehensive analysis of chlorophyll and carotenoid-related genes in celery and six representative species. Significantly, different contents and gene expression patterns were found among three celery varieties. In total, 237 and 290 chlorophyll and carotenoid-related genes were identified in seven species. No notable gene expansion of chlorophyll biosynthesis was detected in examined species. However, the gene encoding ζ-carotene desaturase (ZDS) enzyme in carotenoid was expanded in celery. Comparative genomics and RNA-seq analyses revealed 16 and 5 key genes, respectively, regulating chlorophyll and carotenoid. An intriguing finding is that chlorophyll and carotenoid-related genes were coordinately regulated by transcriptional factors, which could be distinctively classified into positive- and negative-regulation groups. Six *CONSTANS* (*CO*)-like transcription factors co-regulated chlorophyll and carotenoid-related genes were identified in celery. In conclusion, this study provides new insights into the regulation of chlorophyll and carotenoid by transcription factors.

## 1. Introduction

As the main pigment, chlorophyll is located on the thylakoid membrane and plays a central role in light absorption during photosynthesis [1,2]. Actually, chlorophyll is one of the most abundant biological and organic molecules and is central to carbon cycling by solar energy capture and photosynthetic carbon fixation [3,4]. Furthermore, chlorophyll has many other functions, such as delaying aging, producing vitamins, detoxification, and anti-allergy [5,6].

Carotenoids are mainly found in leaves, flowers, fruits and roots of plants [7]. It can attract insects and birds, which help plants spread pollen and seeds [8]. Carotenoids, as antenna pigments, capture light energy and transfer it to chlorophyll, promoting the photochemical process in photosynthesis [9,10]. In addition, carotenoids protect photosynthetic machinery from harmful oxidative light damage caused by strong light [11]. Previous studies also showed that carotenoids had important effects on the human body with antioxidant, immune regulation, and anti-cancer properties [12,13,14,15].

Carotenoids were synthesized by the isoprene pathway and shared a precursor that was called geranylgeranyl pyrophosphate (GGPP) with chlorophyll [3,16]. However, molecular mechanisms regulating GGPP allocation among the two biosynthetic pathways localized in the same subcellular compartment are largely unknown. In vascular plants, chlorophyll and carotenoids are synthesized in chloroplasts, and most enzymes in the synthesis pathway are encoded by nuclear genes [17].

In the chlorophyll biosynthesis pathway, most chlorophyll synthetase genes have been identified from Arabidopsis [18]. The first step is to transfer L-glutamyl-tRNA (Glu-tRNA) to L-glutamic acid (GSA) by glutamyl t-RNA reductase (GluTR). GluTR is encoded by HEMA1, HEMA2 and HEMA3, which have been mapped and studied in Arabidopsis [19,20]. Subsequently, glutamate 1-semialdehyde aminotransferase (GSA-AM) catalyzes GSA to aminolevulinic acid (ALA), which is the rate-limiting step of chlorophyll synthesis. GSA-AM is encoded by two genes, *GSA1* and *GSA2*, in Arabidopsis [21,22]. Secondly, protoporphyrin IX (Proto IX) is catalyzed by Mg chelataseⅠ subunit to produce Mg-protoporphyrin IX (Mg-Proto IX). Mg-Proto IX is catalyzed by Mg-protoporphyrin IX methyltransferase (MgPMT) to form Mg-protoporphyrin IX monomethylester (Mg-PME) in Arabidopsis [23].

In the carotenoid pathway, phytoene synthase (PSY) catalyzes the first step reaction and controls the carbon flow to the carotenoid [24]. Then, the followed consecutive reactions that convert phytoene to lycopene are catalyzed by several enzymes, including phytoene desaturase (PDS), ζ-carotene desaturase (ZDS), ζ-carotene isomerase (ZISO), and carotenoid isomerase (CRTISO) [25,26]. The PDS was overexpressed in Solanum lycopersicum by transgene and it was found that the carotenoid content of the transgene type was two times higher than that of the wild type [27]. Finally, lycopene β-cyclase (LCYb) and lycopene ε-cyclase (LCYe) catalyze the lycopene to form α-carotene and β-carotene [26,28]. In *Brassica napus*, silent LCYe expression resulted in a 2.6–41.7-fold growth in total carotenoid content [29].

Celery (*Apium graveolens* L. 2 *n* = 2 x = 22), grown worldwide, is a popular herb and important vegetable [30,31]. It has many pharmacologically active compounds, such as flavonoids, volatile oils, and unsaturated fatty acids [32,33]. As an *Apiaceae* plant, it has an ‘umbel’ or umbrella-like inflorescence [34]. The *Apiaceae* contains more than 400 genera and about 3820 species [35]. However, comparative and functional genomic studies about *Apiaceae* species have been scarce due to the few genomes released. Until now, the carrot genome has previously been sequenced, which was the first reported genome of *Apiaceae* species [36]. To clarify *Apiaceae* biology and evolution, we produced the high-quality chromosomal-level coriander genome [37,38]. Recently, we also produced a high-quality chromosomal-level genome sequence of celery using PacBio, illumina, and Hi-C technologies. These efforts provide valuable opportunities to identify and study the important functional genes in celery. The aims of this study are to identify functional genes regulating chlorophyll and carotenoid to understand the related biology in celery.

## 2. Results

### 2.1. Comparative Analysis of Chlorophyll and Carotenoid Content in Three Varieties of Celery

**Figure 1:**
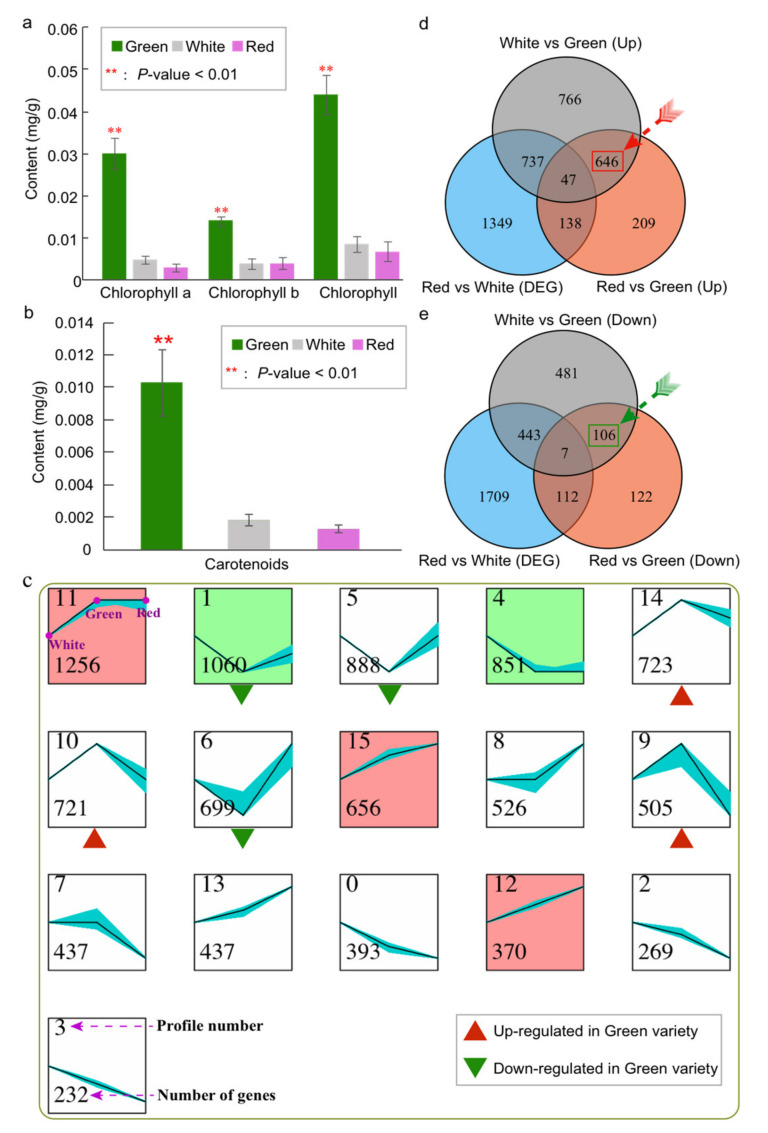
The chlorophyll and carotenoid content, and gene expression analysis of three celery varieties with different petiole colors. (**a**) Content (mg/g) of chlorophyll, chlorophyll a, and chlorophyll b of three celery varieties (green, white, and red). The red asterisk represents significantly different (*p* < 0.01) content between the green and the other two varieties. (**b**) Content (mg/g) of carotenoids of three celery varieties. (**c**). The expression patterns of differentially expressed genes (DEGs) constructed by the STEM program. Each square represents a kind of expression mode. The squares with the same background color indicate a similar expression pattern. The black lines represent the model expression patterns, the blue lines show the expression pattern of individual DEGs. The number in the upper-left corner shows the cluster profile number, and the number in the lower-left corner shows the number of DEGs. The red and green triangles represent the expression pattern of up- and down-regulated genes in the green variety, respectively. (**d**) Venn diagrams show the common and specific up-regulated expressed genes among three varieties. (**e**) Venn diagrams show the common and specific down-regulated expressed genes among three varieties.

Here, the present research is based on three celery varieties with different-colored petioles, including green, white, and red. Firstly, we measured the chlorophyll and carotenoid content among three varieties. The average chlorophyll content in green celery variety (0.0453 mg/g) was 5.4 and 9.6 times more than white (0.0083 mg/g) and red (0.0047 mg/g) varieties, respectively (Figure 1a and Table S1). Specifically, the chlorophyll a and chlorophyll b content in the green variety were all significantly higher than those in white or red varieties (*p*-value < 0.01) (Figure 1a and Table S2). However, no significant difference was found between white and red varieties for chlorophyll and carotenoid content.

Carotenoids, first discovered in carrot [36,39], were 6.8 and 9.8 times (*p*-value < 0.01) more abundant in green celery (0.0116 mg/g) than in white (0.0016 mg/g) and red (0.0011 mg/g) varieties, respectively (Figure 1b, Tables S1 and S2). Similarly, no significant difference was found between white and red varieties. Therefore, these three celery varieties were excellent materials for us to explore the regulation of gene expression related to chlorophyll and carotenoid.

### 2.2. Exploring the Gene Expression Pattern among Three Varieties of Celery

We conducted the expression pattern analysis of these three varieties using the transcriptomic datasets obtained by our laboratory. Based on the expression values of whole-genome genes of three celery varieties, clustering analysis was employed using STEM software. The genes were classified into 16 distinct profiles, and each profile represented a group of genes with the same expression pattern (Figure 1c). There were the most genes (1256) in profile 11, followed by profiles 1 (1060) and 5 (888). Among them, five profiles were significantly enriched (*p*-value < 0.05), and further divided into 2 clusters (profiles 1 and 4; profiles 11, 12, and 15) with different expression trend according to the background color (Figure 1c).

Based on the differences in chlorophyll and carotenoid content among the three varieties, we managed to find profiles that had consistent expression patterns in both white and red celery, while they were not consistent with those in green celery. We found that the gene expression in the green variety was lower than that of the red and white varieties in profiles 1, 5, and 6 (Figure 1c). On the contrary, the gene expression in green was higher than that of red and white varieties in profiles 9, 10, and 14. Therefore, we hypothesized that the genes in these six profiles might be related to the synthesis of celery chlorophyll and carotenoid.

### 2.3. Identification of Differentially Expressed Genes and Functional Enrichment Analysis among Three Varieties

In order to more accurately identify the genes related to chlorophyll and carotenoid, we analyzed the differentially expressed genes (DEGs) among the three varieties. Here, we attempted to identify the DEGs between green vs red celery and green vs white celery, and these genes were not differentially expressed between red and white celery according to the differences in chlorophyll and carotenoid content among the three varieties. By that standard, we detected 646 significantly up-regulated genes and 106 down-regulated genes in green celery compared with red and green varieties (Figure 1d,e).

**Figure 2:**
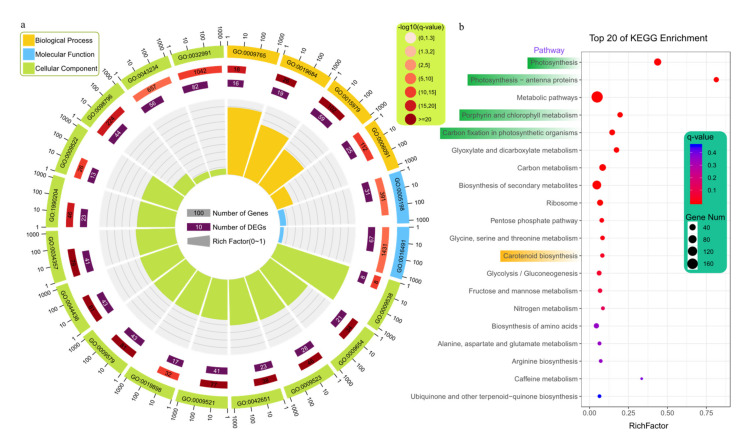
GO and KEGG enrichment analysis (*q*-value < 0.05) of up-regulated genes in green celery compared with the other two celery varieties. (**a**) GO enrichment analysis. The orange, blue, and green colors represent the biological process, molecular function, and cellular component, respectively. The elements from inside to outside of the circle are as follows, the rich factor, the DEG number, All gene number, and the GO accession number. The gradual red color represents –log_10_ (*q*-value) of enrichment analysis. (**b**) The top 20 pathways of KEGG enrichment analysis. The size of dot indicates the number of enriched genes, and the gradual color of the dot corresponds to different *q*-values.

Furthermore, we conducted the functional enrichment analysis of these DEGs using GO and KEGG databases. The results indicated these 646 up-regulated genes in the green variety were significantly enriched (*q*-value < 0.05) in 126 terms, including 57 ‘biological process’, 29 ‘cellular component’, and 40 ‘molecular function’ categories (Figure 2a, Table S3, Figure S1). However, there was no significantly enriched GO term for the down-regulated genes in the green variety (Figure S2). Among the top 20 enriched terms, the terms involved in the biological process mainly were photosynthesis, including light reaction and harvesting. For the cellular component, most terms belonged to thylakoid, photosystem, photosynthetic membrane, and thylakoid membrane. For the molecular function, two terms were involved in the structural molecule and oxidoreductase activity. This indicated that most of the enriched GO terms were related to photosynthesis, indicating that most of these genes up-regulated in green celery were related to photosynthesis. These results were also consistent with the high content of chlorophyll in the green celery variety.

Similarly, we obtained 13 enriched pathways for the up-regulated genes in the green variety using the KEGG database, and no enriched pathway was detected for the down-regulated genes in the green variety (Figure 2b and Table S4). Among these enriched pathways, most of them were also related to photosynthesis and chlorophyll metabolism, which was consistent with the GO enrichment analysis. In addition, the carotenoid pathway was enriched (*q*-value < 0.05) for the up-regulated genes in green celery, which was consistent with the content of carotenoid in the three celery varieties.

### 2.4. Comprehensive Identification of Genes Involved in Chlorophyll and Carotenoid

**Figure 3:**
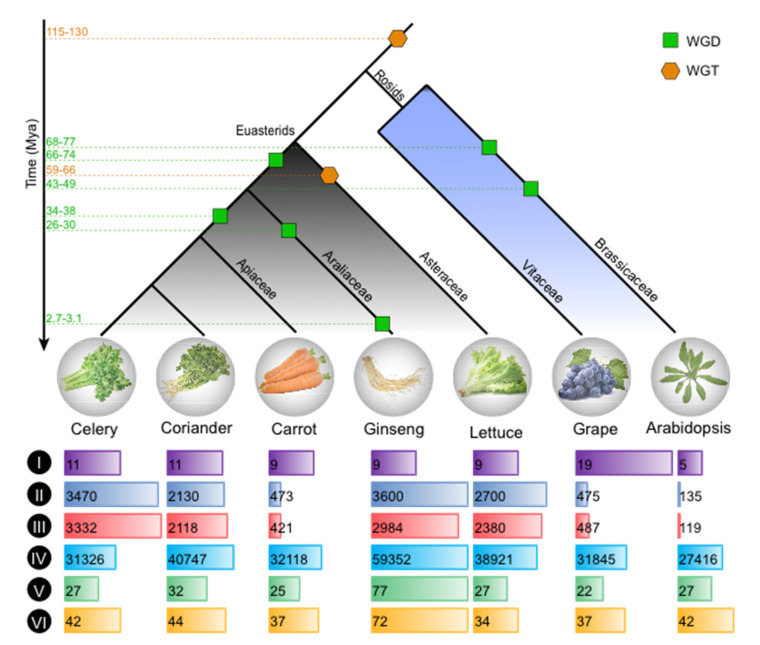
Evolutionary dating, global, and local alignment of genomes. Phylogenetic trees for celery, coriander, carrot, ginseng, lettuce, grape, and Arabidopsis. The green square and orange hexagon represent whole-genome duplication (WGD) and whole-genome triplication (WGT), respectively. The numbers on the dotted line represent the occurred time of each genome duplication event. I, Chromosome number. II, Estimated genome size. III, Assembled genome size. IV, All gene number. V, Chlorophyll biosynthesis gene number. VI, Carotenoids related gene number.

To better explore and comprehensive comparative analysis of genes involved in chlorophyll and carotenoid pathways, we also selected six other representative eudicot species, including two other *Apiaceae* (coriander and carrot), one *Araliaceae* (ginseng: *Panax ginseng*) [40], one *Asteraceae* (lettuce: *Lactuca sativa*) [41], and two Rosids (the botanical model *Arabidopsis thaliana*, and the genome structure model *Vitis vinifera*) (Figure 3). All species used except *Vitis vinifera* (grape) and *Arabidopsis* belonged to the Euasterids.

The chlorophyll biosynthesis pathway is completed by a series of enzymatic reactions (Tables S5 and S6). According to the KEGG database, the sequences of 27 *Arabidopsis* genes contributing to the formation of chlorophyll a and chlorophyll b were used to identify homologous genes in celery and other plants. In total, 27, 32, 25, 77, 27, and 22 chlorophyll biosynthesis genes were identified from the whole genomes of celery, coriander, carrot, ginseng, lettuce, and grape, respectively (Figure 3, Table S6). Similarly, we collected 18 *Arabidopsis* genes involved in carotenoid according to the KEGG pathway, and they further were used as seeds to identify homologous genes in celery and other five species (Figure 3; Tables S7 and S8). The most carotenoid-related genes were identified in ginseng (72), followed by coriander (44), celery (42), carrot (37), grape (37), and lettuce (34).

### 2.5. Phylogenetic and Gene Duplication Analyses

**Figure 4:**
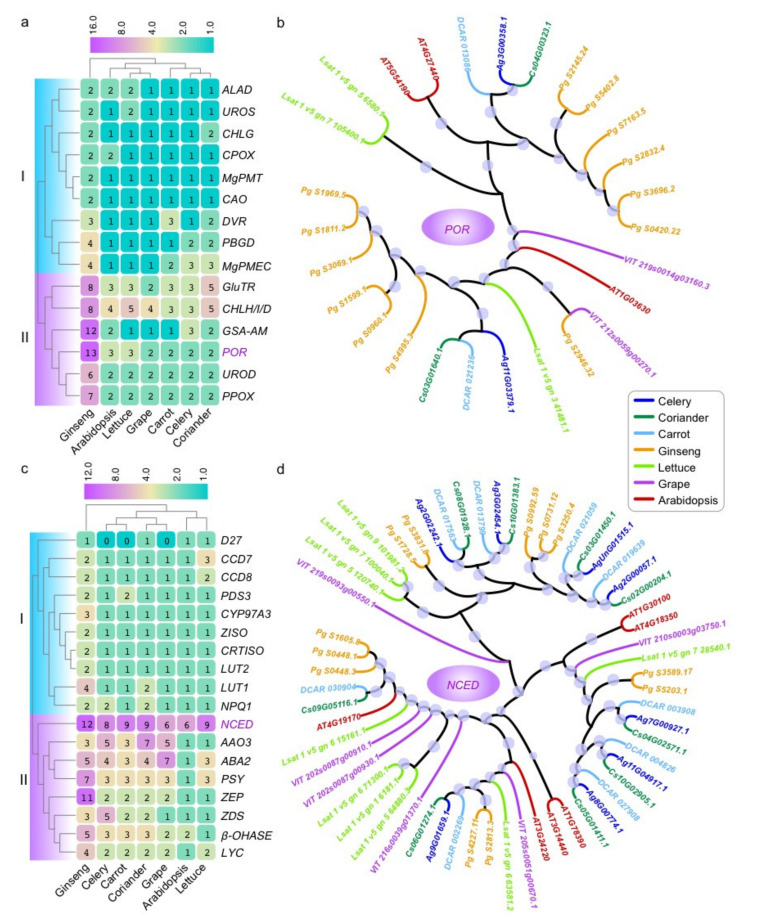
The number and phylogenetic tree of chlorophyll and carotenoids related genes in celery and other six species. (**a**) Heatmap for number of each chlorophyll biosynthesis gene in seven species. (**b**) The maximum-likelihood (ML) tree of *POR* genes was generated based on the amino acid sequences with 1000 bootstrap repeats in seven species. The size of the circle on the branch represents the value of the bootstrap with ranges from 40 to 100. (**c**) Heatmap for number of each carotenoid-related gene in seven species. (**d**) The ML tree of *NCED* genes was generated based on the amino acid sequences with 1000 bootstrap repeats in seven species.

To further explore the evolution of chlorophyll biosynthesis genes, 210 homologous genes encoding 15 enzymes were identified in celery and the other 5 species according to the *Arabidopsis* (Figure 3; Table S6). Based on the number of these genes, we conducted the cluster analysis among seven species, and two groups (I and II) were detected (Figure 4a). In group II, we found there were notably more genes in ginseng than other six species.

Considering shared and specific whole-genome duplication/triplication (Figure 3), assuming no gene loss after their divergence from the last common ancestor, the ratio of gene number among grape, *Arabidopsis*, lettuce, ginseng, carrot, coriander, and celery should be 1:4:3:6:4:4:4 (Figure 3). However, it turned out that in fact most genes did not reach this ratio in multiple species, and most of these genes were lost to varying degrees. This might be due to the loss of genes during evolution after genome duplication. Interestingly, the genes encoding GSA-AM and POR enzymes were expanded in ginseng compared with the grape according to the heatmap and phylogenetic analyses (Figure 4b and Figure S3). There were the most genes encoding several enzymes in ginseng, such as GluTR, CHLH/I/D, GSA-AM, POR, UROD, and so on (Figure 4a,b and Figure S3). Comparatively, there was no notable gene expansion in celery and other examined species for chlorophyll biosynthesis genes.

According to the KEGG pathway, we mainly investigated 18 enzymes involved in the carotenoid. In total, 266 homologous genes encoding these enzymes were identified in celery and the other 5 species according to the *Arabidopsis* (Figure 3; Table S8). Two groups (I and II) were also obtained according to the cluster analysis of the number of these genes (Figure 4c). Compared to the *Arabidopsis*, there were more genes encoding each enzyme in other species, especially for the ginseng in group II. Similar to the chlorophyll biosynthesis genes, each species experienced some degree of gene loss after genome duplication. Interestingly, the genes encoding the ZDS enzyme were expanded in celery compared with the grape, while it was contracted in the other five species. There were the most genes encoding AAO3 enzyme in coriander, ABA2 in grape, PSY and ZEP genes in ginseng (Figure 4c,d and Figure S4). Compared to other enzymes, there were more genes encoding NCED than other enzymes in almost species (Figure 4d).

### 2.6. Expression Analysis of Chlorophyll and Carotenoid Biosynthesis Genes in Celery

**Figure 5:**
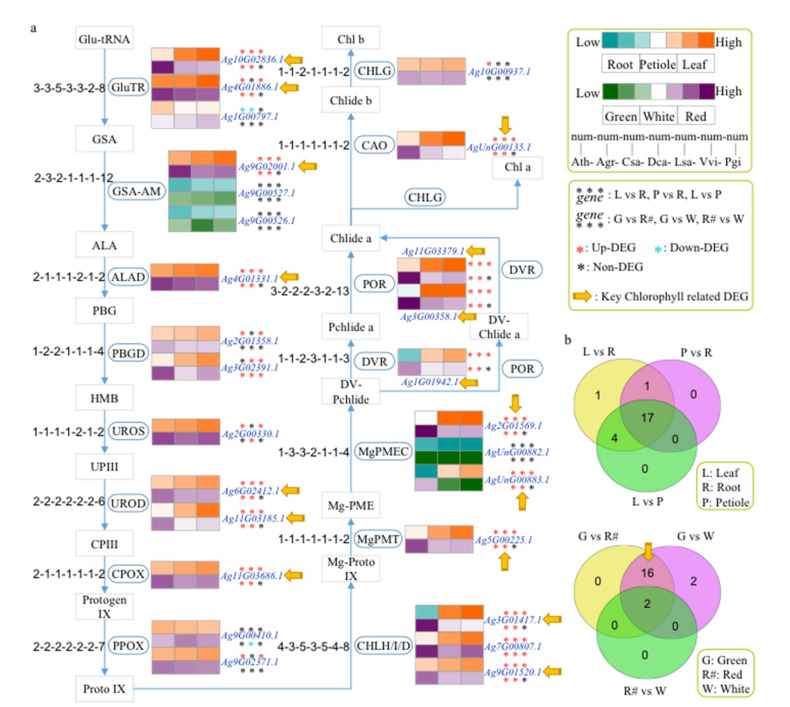
Inferred chlorophyll biosynthesis genes of celery and other 6 species. (**a**) Chlorophyll biosynthesis gene identification in celery and 6 other species. The notation ‘1-1-1-1-1-1-1′ indicates that one gene was identified in Arabidopsis, celery, coriander, carrot, lettuce, grape, and ginseng, respectively. Gene expression was detected in the 3 different tissues and 3 different colors’ varieties of celery. The orange and purple colors indicate high expression levels in different tissues and varieties, respectively. Asterisks represent the up-DEGs (red), down-DEGs (blue), and non-DEGs (black). The yellow arrow represents the key chlorophyll-related DEGs between green and red/white varieties. (**b**) Venn diagrams show differentially expressed genes (DEGs) among three tissues (root, leaf, and petiole) and three varieties (green, red, and white).

Glutamyl tRNA reductase (GluTR) is the first critical rate-limiting reaction in the chlorophyll synthesis pathway. Among 27 chlorophyll biosynthesis genes identified in celery according to the KEGG database, 17 of them were differentially expressed among leaf, root, and petiole tissues (Figure 5a,b and Table S9). Moreover, 16 were all expressed as significantly higher in the green variety than in the red/white varieties. Therefore, these 16 DEGs might play key roles in the synthesis of high chlorophyll content in green celery (Figure 5b). Furthermore, we found that these key DEGs encoded 11 enzymes based on the chlorophyll biosynthesis pathway (Figure 5a and Table S6). The regulatory pathway showed that each node has one or more gene copies in the seven species. There are three genes encoding Glutamate-1-semialdehyde-2,1-aminomutase (*GSA-AM*) in celery, more than in the other five species except for ginseng (Figure 5a).

**Figure 6:**
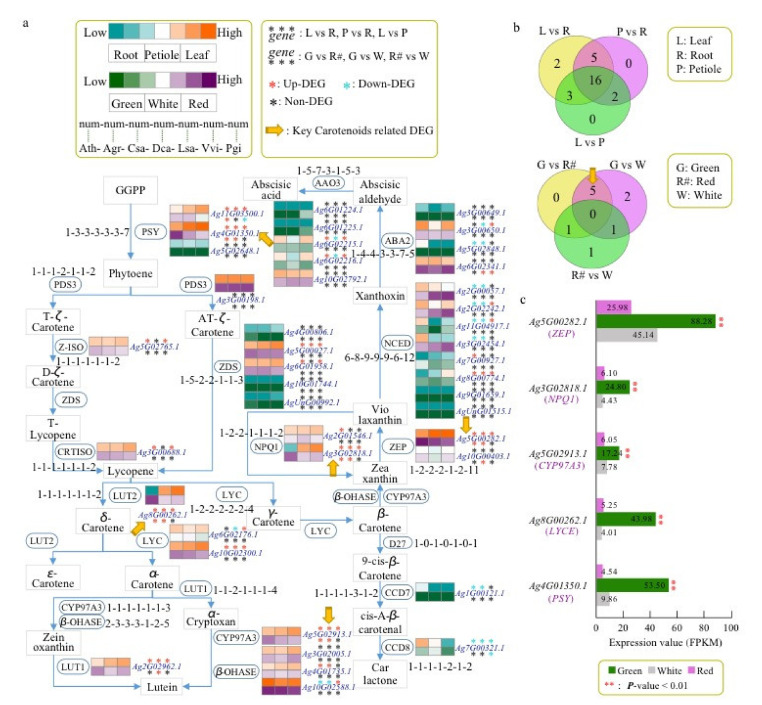
Inferred carotenoid-related genes of celery and other 6 species. (**a**) Carotenoid-related genes identification in celery and other 6 species. The notation ‘1-1-1-1-1-1-1’ indicates that one gene was identified in Arabidopsis, celery, coriander, carrot, lettuce, grape, and ginseng, respectively. Gene expression was detected in the 3 different tissues (root, leaf, and petiole) and 3 different colors’ varieties of celery. The orange and purple colors indicate high expression levels in different tissues and varieties, respectively. Asterisks represent the up-DEGs (red), down-DEGs (blue), and non-DEGs (black). The yellow arrow represents the key carotenoid-related DEGs between green and red/white varieties. (**b**) The Venn diagrams show the DEGs among three tissues (root, leaf, and petiole) and three varieties (green, red, and white). (**c**) The bar plot of expression level (FPKM) of five key carotenoid-related genes in three varieties.

Among all 42 carotenoid-related genes identified in celery, 16 genes were differentially expressed among leaf, root, and petiole tissues (Figure 6a,b and Table S10). Five DEGs (*Ag4G01350.1*, *Ag8G00262.1*, *Ag5G02913.1*, *Ag3G02818.1*, and *Ag5G00282.1*) were all expressed significantly higher in the green variety than in the red/white varieties (Figure 6b,c and Table S10). Therefore, these DEGs might play important roles in the synthesis of high carotenoid content in green celery. Based on the carotenoid pathway, the DEG *Ag4G01350.1* encoded the phytoene synthase (PSY), which condensed two GGPP molecules to produce phytoene, the first compound in the carotenoid pathway (Figure 6a,c). The DEG *Ag8G00262.1* encoded the lycopene epsilon-cyclase (LYCE), which catalyzed the synthesis of δ-carotene from lycopene. The δ-carotene was further formed into α-carotene by the action of lycopene beta-cyclase (LYCB). The other three differentially expressed genes encoded a cytochrome P450 family member (CYP97A3), non-photochemical quenching 1 (NPQ1) and zeaxanthin epoxidase (ZEP), and these enzymes were mainly involved in the synthesis of downstream carotenoid-related substances. Therefore, we believe that the first two genes, *Ag4G01350.1* and *Ag8G00262.1*, played a very important role in the accumulation of carotene in green celery.

Although the corresponding encoding genes of these two enzymes were not significantly differentially expressed between the three cultivars of celery, the genes encoding ZDS enzymes were significantly expanded in celery. A total of five *ZDS* genes were detected in celery, which was more than that in the other six species (Figure 6a). Only two *ZDS* genes were found in the other two *Apiaceae* species (carrot and coriander). Based on the phylogenetic tree, we found that three *ZDS* genes (*Ag4G00806.1*, *Ag10G01744.1*, *AgUnG00992.1*) in celery had no corresponding genes in carrot and coriander, suggesting that they might be duplicated genes produced during genome duplication. Therefore, the above findings showed that the *ZDS* enzyme might play important an role in celery carotenoid.

### 2.7. Regulatory Networks Construction for Chlorophyll and Carotenoid-Related Genes with Transcription Factors

**Figure 7:**
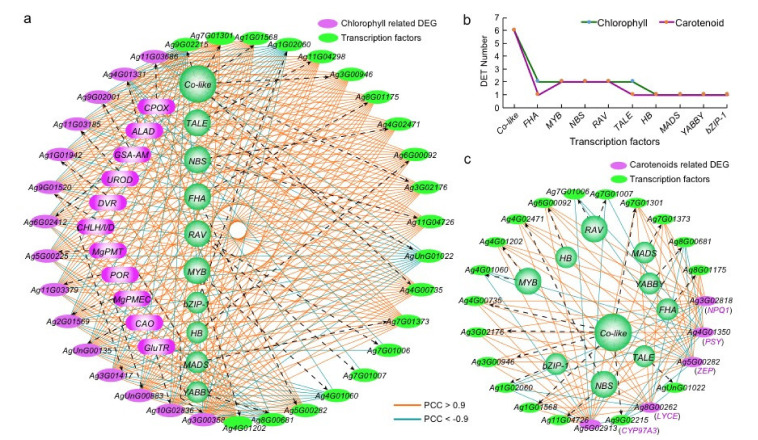
The interaction network between key chlorophyll and carotenoid-related DEGs and differently expressed TFs between green variety and both red and white varieties. (**a**) The interaction network between key chlorophyll-related DEGs (purple oval) and differently expressed TFs (DETs, green oval). The orange and blue lines represent the Pearson correlation coefficient (PCC) values larger than 0.9 (positively regulated relationship) or lower than −0.9 (negatively regulated relationship), respectively. (**b**) The number of DETs involved in the network of chlorophyll and carotenoid in celery. (**c**) The interaction network between key carotenoid-related DEGs and DETs.

We constructed regulatory networks between TFs and the 16 key chlorophyll-related DEGs based on Pearson correlation coefficients (PCCs). All the differentially expressed TFs (DETs) between green celery and the other two colored varieties were used, with 20 DETs and 15 (out of 16) key chlorophyll-related DEGs together forming this network (Figure 7a). The DETs in the network were classified into 10 categories, of which *CO*-like ones were the most (6), followed by *MYB*, *NBS*, *ABI3*/*VP1* (*RAV*), and so on (Figure 7b and Table S11). More network connections between DEGs and DETs show positive (194 with PCC > 0.9) than negative regulation (64 with PCC < −0.9) (Table S12). Twenty DETs fell into two distinct groups, including thirteen positive- and seven negative-regulation groups (Figure 7a). Interestingly, some homologous DETs, such as six *CO-like* TFs, five of them performed positive regulation of chlorophyll biosynthesis, while the *Ag9G02215* was just reverse (Figure 7a).

An inferred regulatory network involves 18 DETs and 5 key carotenoid-related DEGs (Figure 7c). The DETs could be classified into 10 categories, of which *CO*-like was the most (6), followed by *MYB*, *NBS* and *ABI3*/*VP1* (*RAV*) (Figure 7b, Table S11). Intriguingly, just like the chlorophyll network, the carotenoid synthesis network DETs formed 11 positive-regulation and 7 negative-regulation groups (Figure 7c). Further, the carotenoid DETs are all contained in the chlorophyll DETs (Figure 6 and Figure 7; Table S11), implicating the same positive or negative regulation and showing highly coordinated regulation of the two pigments by the same set of transcriptional factors. Some homologous DETs, e.g., *Ag7G01301* and *Ag9G02215*, respectively perform positive and negative regulation of carotenoid, coordinated to that in the chlorophyll biosynthesis.

## 3. Discussion

Chlorophyll is an important pigment involved in plant photosynthesis. It captures light energy and drives electrons to transfer to the reaction center [42,43]. The mutation of chlorophyll biosynthesis genes can lead to the loss or decrease in related enzyme activities, resulting in the decrease in chlorophyll content and the etiolation of leaves. Finally, it will lead to a decrease in photosynthetic efficiency, slow growth and even death [44,45]. In higher plants, chlorophyll mainly includes chlorophyll a and chlorophyll b, and most enzymes required for chlorophyll biosynthesis have been detected [46,47]. Twenty-seven genes encoding these enzymes have been cloned in Arabidopsis, providing an important reference for the identification of chlorophyll synthesis genes in the other species [48].

Inhibition of carotenoid or chlorophyll synthesis to inhibit plant photosynthesis is one of the action mechanisms of herbicides [49,50]. Carotenoid or chlorophyll biosynthesis inhibitors can cause albino leaves and even lead to death. The main reason for this phenomenon is the inhibition of carotenoid or chlorophyll biosynthesis in plants [3,51]. By transplanting genes related to chloroplast and carotenoid synthesis into plants, it is possible to make plants resistant to different types of herbicides [10]. Therefore, genes identified in this study are of great value for breeding new herbicide-resistant plants or developing new herbicides.

Transcription factors (TFs) play important roles in regulating carotenoid and chlorophyll-related genes [52,53]. For example, the down-regulation of SlMYB72 played an important role in changing the expression level of genes related to chlorophyll, carotenoid and flavonoid in *Solanum lycopersicum* [54]. In this study, we constructed regulatory networks between differentially expressed TFs (DETs) and the key chlorophyll- and carotenoid-related DEGs. Most DETs in the network were *CO*-like, *MYB*, *NBS*, *ABI3*/*VP1* (*RAV*), and they performed positive or negative regulation of chlorophyll and carotenoid-related genes in celery.

An intriguing finding from the present study is that chlorophyll and carotenoids are regulated by mostly shared but differentially expressed TFs (DETs) in the same regulatory pattern. Carotenoids are integral constituents of the thylakoid membrane and are usually associated intimately with both antenna and reaction center pigment proteins [10]. The light absorbed by carotenoids is transferred to chlorophyll for photosynthesis; because of this role, they are called accessory pigments. Carotenoids are tetraterpenes and the phytol side chain of chlorophyll is terpene-derived, therefore, they have some common precursors such as geranylgeranyl diphosphate (GGPP) [18,55]. Here, we found that the DETs, while regulating carotenoid or chlorophyll, could be distinctively classified into two groups, with one group always performing positive regulation and the other negative regulation.

A high-quality chromosome-level celery reference genome and transcriptome, together with genomic data for carrot and coriander, provides abundant resources for both fundamental and applied research into *Apiaceae* plants [36,37,38]. Two sequential WGDs leading to the formation of celery and other *Apiaceae* genomes exerted a great impact on gene regulatory networks and may have contributed to the diversification of the *Apiaceae* [35].

In this study, the molecular basis of celery chlorophyll and carotenoid regulatory networks were comprehensively analyzed in combination with transcriptome analysis. Several key genes related to these pathways were identified in celery and their expression was explored between different tissues and varieties, laying a solid foundation for dissecting genetic mechanisms associated with photosynthesis.

## 4. Materials and Methods

### 4.1. Genomic Data Retrieval

The gene sequences and other related datasets of celery used in this study can be downloaded from database of our laboratory (BIO2DB: http://bio2db.com (accessed on 26 July 2022)) [56]. The sequences of coriander (v1.0) were downloaded from Coriander Genomics Database (CGDB) [37]. The sequences of Arabidopsis were downloaded from the Arabidopsis Information resource (http://www.arabidopsis.org/ (accessed on 26 July 2022)). The sequences of ginseng were obtained from the previous report [40]. The sequences of carrot (v2.0), lettuce (v5.0), and grape (Genoscope.12X) were downloaded from Phytozome database (http://www.phytozome.net/ (accessed on 26 July 2022)) [36,41,57].

### 4.2. Detection of Chlorophyll and Carotenoid Content

In the study, a total of 0.5 g stems of three varieties (green celery, red celery and white celery) were taken for the determination of chlorophyll and carotenoid content, and each celery variety was repeated three times. Then, 5 mL of 95% ethanol was used for extraction at 4 °C. The content of chlorophyll and carotenoid was measured by spectrophotometry from the petiole of three celery varieties.

### 4.3. Chlorophyll and Carotenoid-Related Genes Identification

Firstly, the genes of Arabidopsis related to chlorophyll and carotenoid were retrieved according to the KEGG database [58]. Then, these genes were used to identify homologous genes in celery and other plants, including coriander, carrot, ginseng, lettuce, and grape, using the Blastp program with the parameters as e-value < 1 × 10^−5^, identify > 50%, score > 100 according to the previous reports [59,60].

### 4.4. Phylogenetic Tree Construction

The phylogenetic trees were constructed using the amino acid sequences of the chlorophyll and carotenoid genes from celery and six other species by MEGA X software [61]. The neighbor-joining (NJ) method was used with 1000 bootstrap replicates. Evolutionary distance was calculated using the Poisson correction model. The evolutionary tree was further beautified using the iTOL program [62].

### 4.5. Gene Expression Data Collection and Substance Content Measurement

The transcriptomic datasets were obtained from our laboratory, which contained three celery samples with different-colored petiole varieties, including green celery ‘Ventura’, white celery ‘Baiqin’, and red celery ‘Hongqin’. In addition, the RNA-seq dataset from three tissues (root, petiole, and leaf) of green celery ‘Ventura’ were used for gene expression analysis. All the samples had three biological replicates, and all the datasets were available download from the publicly accessible database with the accession numbers CRA001996 and CRA001997 according to the previous report [56]. Furthermore, we measured the content of chlorophyll and carotenoid using spectrophotometry from the petiole of these three celery genotypes for comparative analysis.

### 4.6. Gene Expression Quantity, Cluster, and Enrichment Analysis

The Cufflinks program was used for calculating gene expression with default parameters, and the expression was normalized as the expected number of Fragments Per Kilobase of transcript sequence per Millions base pairs sequenced (FPKM) [63]. The DEGs analyses were conducted using DESeq2 software with *p*-adj < 0.05 and the absolute value of fold-change >2 [64]. The hierarchical clustering heat map and Venn diagram were visualized using TBtools software (v1.09876) [65]. The expression patterns of genes in different varieties or tissues were obtained using the STEM software (v1.3.13) [66]. The number of model profiles was set to 50, and other settings used the default parameters. The GO and KEGG enrichment analysis was performed using the OmicShare tools (http://www.omicshare.com/tools (accessed on 26 July 2022)), and *q*-values less than 0.05 were thought to be significantly enriched in this study.

### 4.7. Interaction Network Construction

All the transcription factors (TFs) were identified using the Pfam database (http://pfam.sanger.ac.uk/ (accessed on 26 July 2022)) with E-value <1 × 10^−4^ according to the previous reports [67,68,69]. Pearson’s correlation coefficients (PCCs) between TFs and DEGs were calculated using in-house Perl scripts according to the gene expression value of three different color’s celery varieties. The positive and negative regulatory relationships were estimated as the PCC > 0.90 and PCC < −0.90, respectively [70,71]. The interaction networks between pathway-related genes and TFs were constructed using Cytoscape software (https://cytoscape.org/ (accessed on 26 July 2022)).

## Data Availability

The datasets analyzed during the current study are available in the Beijing Institute of Genomics (BIG) Data Center under accession numbers CRA001993, CRA001996, CRA001997 that are publicly accessible at http://bigd.big.ac.cn/gsa (accessed on 26 July 2022). The assembled celery genome and related dataset also can be downloaded from our Apiaceae genome database (http://cgdb.bio2db.com (accessed on 26 July 2022)). All materials and related data in this study are provided in the supplementary files. Other data are available upon request to the corresponding author.

## Supplementary Materials

The following supporting information can be downloaded at: https://www.mdpi.com/article/10.3390/ijms23168986/s1.

## References

[1] Chen M. (2014). Chlorophyll modifications and their spectral extension in oxygenic photosynthesis. Annu. Rev. Biochem..

[2] Das A., Guyer L., Hortensteiner S. (2018). Chlorophyll and Chlorophyll Catabolite Analysis by HPLC. Methods Mol. Biol..

[3] Kim S., Schlicke H., Van Ree K., Karvonen K., Subramaniam A., Richter A., Grimm B., Braam J. (2013). Arabidopsis chlorophyll biosynthesis: An essential balance between the methylerythritol phosphate and tetrapyrrole pathways. Plant Cell.

[4] Reinbothe C., El Bakkouri M., Buhr F., Muraki N., Nomata J., Kurisu G., Fujita Y., Reinbothe S. (2010). Chlorophyll biosynthesis: Spotlight on protochlorophyllide reduction. Trends Plant Sci.

[5] Solymosi K., Mysliwa-Kurdziel B. (2017). Chlorophylls and their Derivatives Used in Food Industry and Medicine. Mini Rev. Med. Chem..

[6] Chen K., Roca M. (2018). Cooking effects on chlorophyll profile of the main edible seaweeds. Food Chem..

[7] Kloer D.P., Schulz G.E. (2006). Structural and biological aspects of carotenoid cleavage. Cell. Mol. Life Sci..

[8] Ito M., Yamano Y., Tode C., Wada A. (2009). Carotenoid synthesis: Retrospect and recent progress. Arch. Biochem. Biophys..

[9] Othman R., Mohd Zaifuddin F.A., Hassan N.M. (2014). Carotenoid biosynthesis regulatory mechanisms in plants. J. Oleo Sci..

[10] Ruiz-Sola M.A., Rodriguez-Concepcion M. (2012). Carotenoid biosynthesis in Arabidopsis: A colorful pathway. Arab. Book.

[11] Llorente B. (2016). Regulation of Carotenoid Biosynthesis in Photosynthetic Organs. Subcell. Biochem..

[12] Kerfeld C.A., Melnicki M.R., Sutter M., Dominguez-Martin M.A. (2017). Structure, function and evolution of the cyanobacterial orange carotenoid protein and its homologs. New Phytol..

[13] Schweiggert R.M., Carle R. (2017). Carotenoid deposition in plant and animal foods and its impact on bioavailability. Crit. Rev. Food Sci. Nutr..

[14] Bohn T. (2019). Determinants and Determination of Carotenoid Bioavailability from Infant Food Formulas and Adult Nutritionals Including Liquid Dairy Products. J. AOAC Int..

[15] Rodriguez-Concepcion M., Avalos J., Bonet M.L., Boronat A., Gomez-Gomez L., Hornero-Mendez D., Limon M.C., Melendez-Martinez A.J., Olmedilla-Alonso B., Palou A. (2018). A global perspective on carotenoids: Metabolism, biotechnology, and benefits for nutrition and health. Prog. Lipid Res..

[16] Sun T., Yuan H., Cao H., Yazdani M., Tadmor Y., Li L. (2018). Carotenoid Metabolism in Plants: The Role of Plastids. Mol. Plant.

[17] McGhie T.K., Ainge G.D. (2002). Color in fruit of the genus actinidia: Carotenoid and chlorophyll compositions. J. Agric. Food Chem..

[18] Lohr M., Im C.S., Grossman A.R. (2005). Genome-based examination of chlorophyll and carotenoid biosynthesis in *Chlamydomonas reinhardtii*. Plant Physiol..

[19] McCormac A.C., Fischer A., Kumar A.M., Söll D., Terry M.J. (2001). Regulation of *HEMA1* expression by phytochrome and a plastid signal during de-etiolation in Arabidopsis thaliana. Plant J..

[20] Ujwal M.L., McCormac A.C., Goulding A., Kumar A.M., Söll D., Terry M.J. (2002). Divergent regulation of the *HEMA* gene family encoding glutamyl-tRNA reductase in *Arabidopsis thaliana*: Expression of *HEMA2* is regulated by sugars, but is independent of light and plastid signalling. Plant Mol. Biol..

[21] Ilag L.L., Kumar A.M., Söll D. (1994). Light regulation of chlorophyll biosynthesis at the level of 5-aminolevulinate formation in Arabidopsis. Plant Cell.

[22] Toyokura K., Yamaguchi K., Shigenobu S., Fukaki H., Tatematsu K., Okada K. (2015). Mutations in Plastidial 5-Aminolevulinic Acid Biosynthesis Genes Suppress a Pleiotropic Defect in Shoot Development of a Mitochondrial GABA Shunt Mutant in Arabidopsis. Plant Cell Physiol..

[23] Richter A.S., Wang P., Grimm B. (2016). Arabidopsis Mg-Protoporphyrin IX Methyltransferase Activity and Redox Regulation Depend on Conserved Cysteines. Plant Cell Physiol..

[24] Qin G., Gu H., Ma L., Peng Y., Deng X., Chen Z., Qu L. (2007). Disruption of phytoene desaturase gene results in albino and dwarf phenotypes in *Arabidopsis* by impairing chlorophyll, carotenoid, and gibberellin biosynthesis. Cell Res..

[25] Busch M., Seuter A., Hain R. (2002). Functional analysis of the early steps of carotenoid biosynthesis in tobacco. Plant Physiol..

[26] DellaPenna D., Pogson B.J. (2006). Vitamin synthesis in plants: Tocopherols and carotenoids. Annu. Rev. Plant Biol..

[27] Gao M., Qu H., Gao L., Chen L., Sebastian R.S., Zhao L. (2015). Dissecting the mechanism of *Solanum lycopersicum* and *Solanum chilense* flower colour formation. Plant Biol..

[28] Chen X., Han H., Jiang P., Nie L., Bao H., Fan P., Lv S., Feng J., Li Y. (2011). Transformation of beta-lycopene cyclase genes from *Salicornia europaea* and *Arabidopsis* conferred salt tolerance in *Arabidopsis* and tobacco. Plant Cell Physiol..

[29] Yu B., Lydiate D.J., Young L.W., Schafer U.A., Hannoufa A. (2008). Enhancing the carotenoid content of *Brassica napus* seeds by downregulating lycopene epsilon cyclase. Transgenic Res..

[30] Browers M.A., Orton T.J., Bajaj Y.P.S. (1986). Celery (*Apium graveolens* L.). Crops I.

[31] Li M., Wang F., Xu Z., Jiang Q., Ma J., Tan G., Xiong A. (2014). High throughput sequencing of two celery varieties small RNAs identifies microRNAs involved in temperature stress response. BMC Genom..

[32] Lin L., Lu S., Harnly J. (2007). Detection and quantification of glycosylated flavonoid malonates in celery, Chinese celery, and celery seed by LC-DAD-ESI/MS. J. Agric. Food Chem..

[33] Sellami I.H., Bettaieb I., Bourgou S., Dahmani R., Limam F., Marzouk B. (2012). Essential oil and aroma composition of leaves, stalks and roots of celery (*Apium*
*graveolens* var. dulce) from Tunisia. J. Essent. Oil Res..

[34] Fu N., Wang Q., Shen H. (2013). De novo assembly, gene annotation and marker development using Illumina paired-end transcriptome sequences in celery (*Apium graveolens* L.). PLoS ONE.

[35] Plunkett G.M., Pimenov M.G., Reduron J.P., Kljuykov E.V., van Wyk B.E., Ostroumova T.A., Henwood M.J., Tilney P.M., Spalik K., Watson M.F., Kadereit J.W., Bittrich V. (2018). Flowering Plants. Eudicots: Apiales, Gentianales (except Rubiaceae).

[36] Iorizzo M., Ellison S., Senalik D., Zeng P., Satapoomin P., Huang J., Bowman M., Iovene M., Sanseverino W., Cavagnaro P. (2016). A high-quality carrot genome assembly provides new insights into carotenoid accumulation and asterid genome evolution. Nat. Genet..

[37] Song X., Nie F., Chen W., Ma X., Gong K., Yang Q., Wang J., Li N., Sun P., Pei Q. (2020). Coriander Genomics Database: A genomic, transcriptomic, and metabolic database for coriander. Hortic. Res..

[38] Song X., Wang J., Li N., Yu J., Meng F., Wei C., Liu C., Chen W., Nie F., Zhang Z. (2020). Deciphering the high-quality genome sequence of coriander that causes controversial feelings. Plant Biotechnol. J..

[39] Just B.J., Santos C.A., Yandell B.S., Simon P.W. (2009). Major QTL for carrot color are positionally associated with carotenoid biosynthetic genes and interact epistatically in a domesticated x wild carrot cross. Theor. Appl. Genet..

[40] Kim N.H., Jayakodi M., Lee S.C., Choi B.S., Jang W., Lee J., Kim H.H., Waminal N.E., Lakshmanan M., van Nguyen B. (2018). Genome and evolution of the shade-requiring medicinal herb Panax ginseng. Plant Biotechnol. J..

[41] Reyes-Chin-Wo S., Wang Z., Yang X., Kozik A., Arikit S., Song C., Xia L., Froenicke L., Lavelle D.O., Truco M.J. (2017). Genome assembly with in vitro proximity ligation data and whole-genome triplication in lettuce. Nat. Commun..

[42] Johnson M.P. (2016). Photosynthesis. Essays Biochem..

[43] Kume A., Akitsu T., Nasahara K.N. (2018). Why is chlorophyll b only used in light-harvesting systems?. J. Plant Res..

[44] Li N., Jia J., Xia C., Liu X., Kong X. (2013). Characterization and mapping of novel chlorophyll deficient mutant genes in durum wheat. Breed. Sci..

[45] Ohmiya A., Hirashima M., Yagi M., Tanase K., Yamamizo C. (2014). Identification of genes associated with chlorophyll accumulation in flower petals. PLoS ONE.

[46] Chatterjee A., Kundu S. (2015). Revisiting the chlorophyll biosynthesis pathway using genome scale metabolic model of *Oryza sativa japonica*. Sci. Rep..

[47] Steccanella V., Hansson M., Jensen P.E. (2015). Linking chlorophyll biosynthesis to a dynamic plastoquinone pool. Plant Physiol. Biochem..

[48] Nagata N., Tanaka R., Satoh S., Tanaka A. (2005). Identification of a vinyl reductase gene for chlorophyll synthesis in *Arabidopsis thaliana* and implications for the evolution of *Prochlorococcus* species. Plant Cell.

[49] Burns E.R., Buchanan G.A., Carter M.C. (1971). Inhibition of carotenoid synthesis as a mechanism of action of amitrole, dichlormate, and pyriclor. Plant Physiol..

[50] Frosch S., Jabben M., Bergfeld R., Kleinig H., Mohr H. (1979). Inhibition of carotenoid biosynthesis by the herbicide SAN 9789 and its consequences for the action of phytochrome on plastogenesis. Planta.

[51] Nisar N., Li L., Lu S., Khin N.C., Pogson B.J. (2015). Carotenoid metabolism in plants. Mol. Plant.

[52] Luo F., Cai J.H., Kong X.M., Zhou Q., Zhou X., Zhao Y.B., Ji S.J. (2019). Transcriptome profiling reveals the roles of pigment mechanisms in postharvest broccoli yellowing. Hortic. Res..

[53] Zhou D., Shen Y., Zhou P., Fatima M., Lin J., Yue J., Zhang X., Chen L.Y., Ming R. (2019). Papaya CpbHLH1/2 regulate carotenoid biosynthesis-related genes during papaya fruit ripening. Hortic. Res..

[54] Wu M., Xu X., Hu X., Liu Y., Cao H., Chan H., Gong Z., Yuan Y., Luo Y., Feng B. (2020). SlMYB72 Regulates the Metabolism of Chlorophylls, Carotenoids, and Flavonoids in Tomato Fruit. Plant Physiol..

[55] Taiz L., Zeman F. (2010). Photosynthesis: The light reaction. Plant Physiol..

[56] Song X., Sun P., Yuan J., Gong K., Li N., Meng F., Zhang Z., Li X., Hu J., Wang J. (2021). The celery genome sequence reveals sequential paleo-polyploidizations, karyotype evolution and resistance gene reduction in apiales. Plant Biotechnol. J..

[57] Jaillon O., Aury J.M., Noel B., Policriti A., Clepet C., Casagrande A., Choisne N., Aubourg S., Vitulo N., Jubin C. (2007). French-Italian Public Consortium for Grapevine Genome, C. The grapevine genome sequence suggests ancestral hexaploidization in major angiosperm phyla. Nature.

[58] Mao X., Cai T., Olyarchuk J.G., Wei L. (2005). Automated genome annotation and pathway identification using the KEGG Orthology (KO) as a controlled vocabulary. Bioinformatics.

[59] Duan W., Huang Z., Song X., Liu T., Liu H., Hou X., Li Y. (2016). Comprehensive analysis of the polygalacturonase and pectin methylesterase genes in Brassica rapa shed light on their different evolutionary patterns. Sci. Rep..

[60] Song X., Wang J., Sun P., Ma X., Yang Q., Hu J., Sun S., Li Y., Yu J., Feng S. (2020). Preferential gene retention increases the robustness of cold regulation in Brassicaceae and other plants after polyploidization. Hortic. Res..

[61] Kumar S., Stecher G., Li M., Knyaz C., Tamura K. (2018). MEGA X: Molecular Evolutionary Genetics Analysis across Computing Platforms. Mol. Biol. Evol..

[62] Letunic I., Bork P. (2019). Interactive Tree of Life (iTOL) v4: Recent updates and new developments. Nucleic Acids Res..

[63] Trapnell C., Williams B.A., Pertea G., Mortazavi A., Kwan G., van Baren M.J., Salzberg S.L., Wold B.J., Pachter L. (2010). Transcript assembly and quantification by RNA-Seq reveals unannotated transcripts and isoform switching during cell differentiation. Nat. Biotechnol..

[64] Anders S., Huber W. (2010). Differential expression analysis for sequence count data. Genome Biol..

[65] Chen C., Chen H., Zhang Y., Thomas H.R., Frank M.H., He Y., Xia R. (2020). TBtools: An Integrative Toolkit Developed for Interactive Analyses of Big Biological Data. Mol. Plant.

[66] Ernst J., Bar-Joseph Z. (2006). STEM: A tool for the analysis of short time series gene expression data. BMC Bioinform..

[67] Finn R.D., Bateman A., Clements J., Coggill P., Eberhardt R.Y., Eddy S.R., Heger A., Hetherington K., Holm L., Mistry J. (2014). Pfam: The protein families database. Nucleic Acids Res..

[68] Wu P., Song X., Wang Z., Duan W., Hu R., Wang W., Li Y., Hou X. (2016). Genome-wide analysis of the *BES1* transcription factor family in Chinese cabbage (*Brassica rapa* ssp. pekinensis). Plant Growth Regul..

[69] Song X., Ma X., Li C., Hu J., Yang Q., Wang T., Wang L., Wang J., Guo D., Ge W. (2018). Comprehensive analyses of the *BES1* gene family in *Brassica napus* and examination of their evolutionary pattern in representative species. BMC Genom..

[70] Song X., Liu G., Huang Z., Duan W., Tan H., Li Y., Hou X. (2016). Temperature expression patterns of genes and their coexpression with LncRNAs revealed by RNA-Seq in non-heading Chinese cabbage. BMC Genom..

[71] Song X., Liu T., Duan W., Ma Q., Ren J., Wang Z., Li Y., Hou X. (2014). Genome-wide analysis of the *GRAS* gene family in Chinese cabbage (*Brassica rapa* ssp. pekinensis). Genomics.

