# Supplementary material for: Transcriptomics and Genomics Analysis Uncover the Differentially Expressed Chlorophyll and Carotenoid-Related Genes in Celery
Source: Int J Mol Sci. 2022 Aug 12;23(16):8986. doi: 10.3390/ijms23168986 (PMC9409461; doi:10.3390/ijms23168986)
Supplement: Supplementary file 1 [file ijms-23-08986-s001.zip › Supplementary Figures S1-S4.pdf]

Supplementary Figures S1–S4

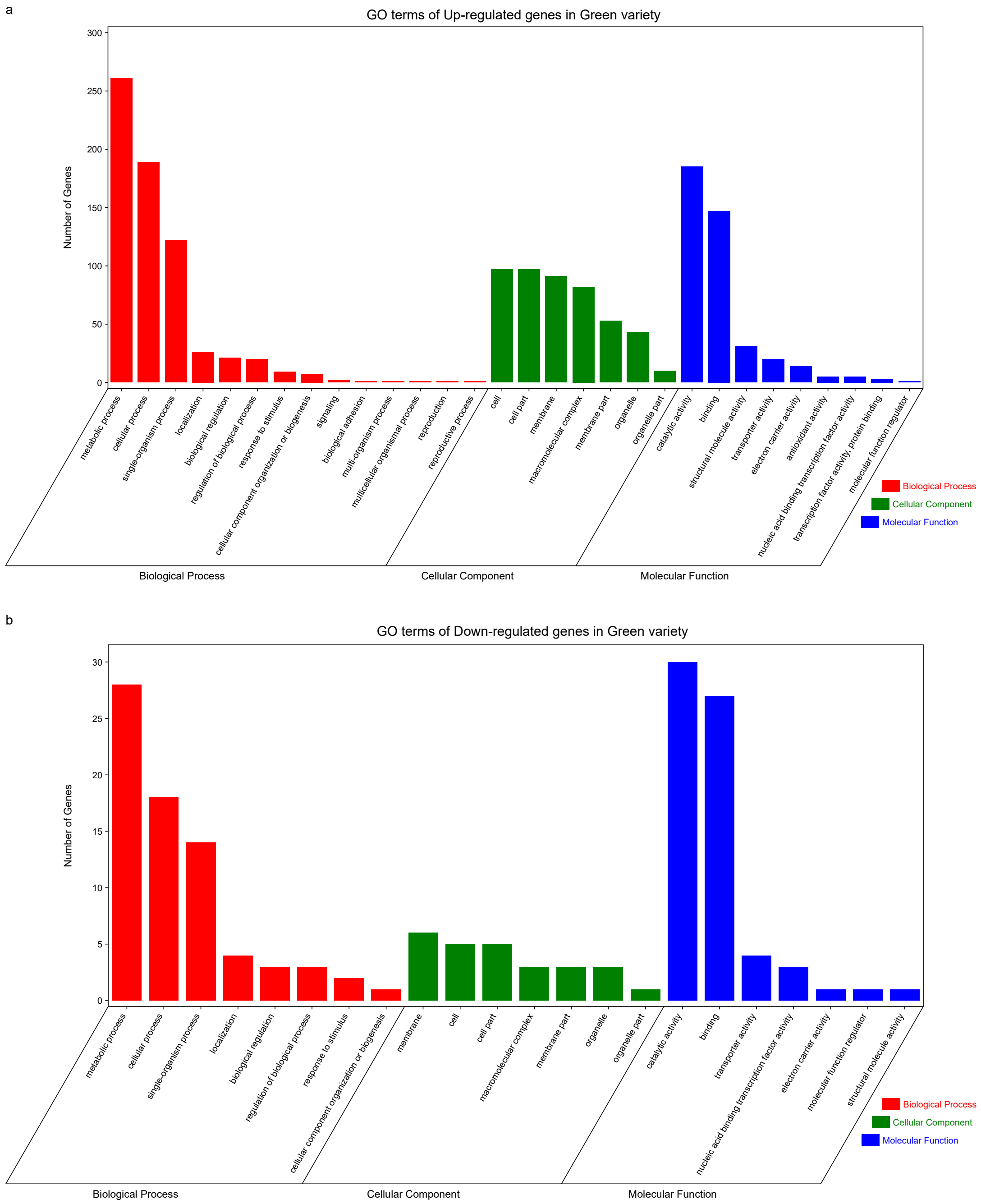

Figure S1. The histogram of GO annotation for differentially expressed genes (DEGs) in green celery comparing with other two varieties. (a) GO terms for up-regulated genes in green celery. (b) GO terms for down-regulated genes in green celery.

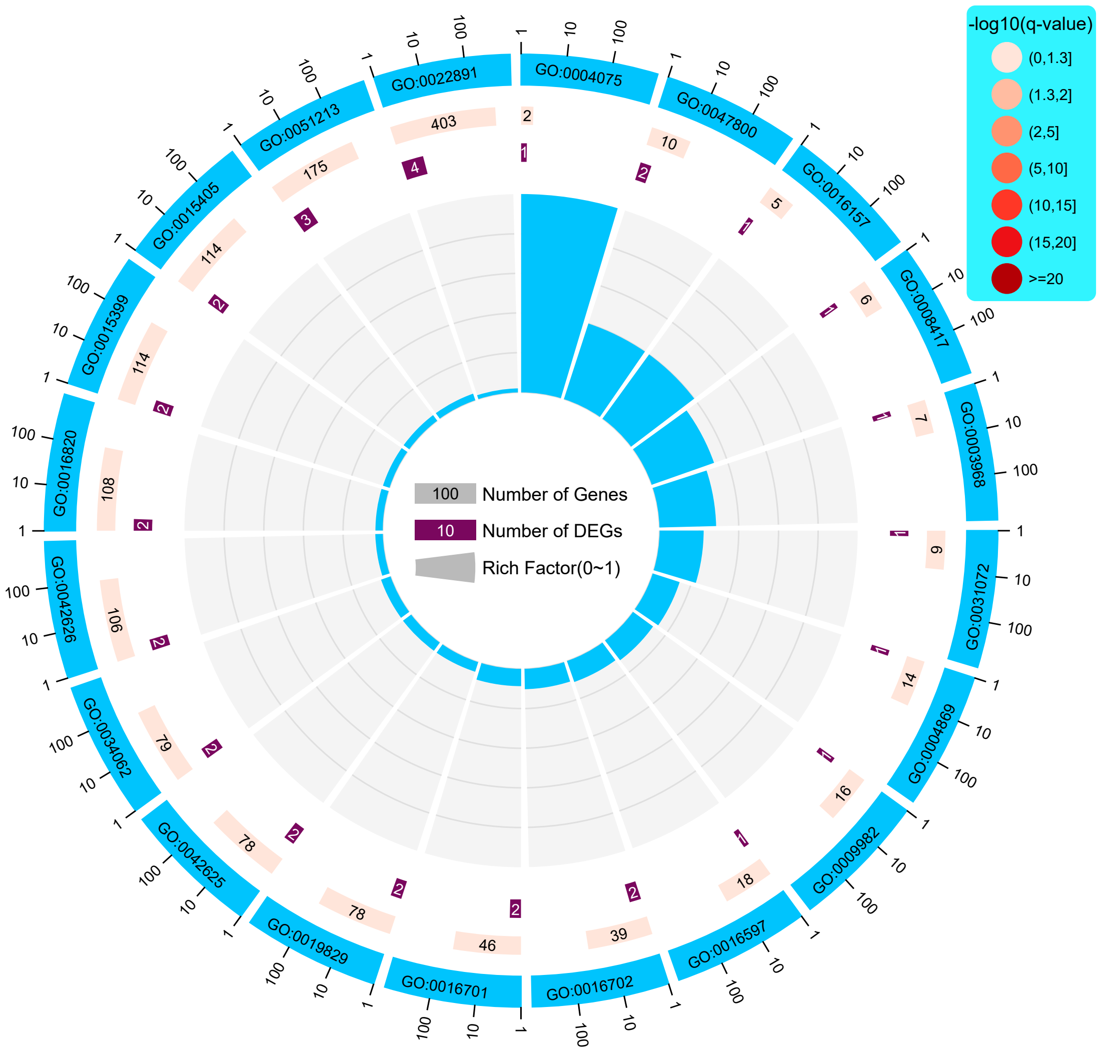

Figure S2. GO enrichment analysis (*q*-value < 0.05) of down-regulated genes in green celery comparing with other two celery varieties. The elements from inside to outside of the circle are as follows, the rich factor, the DEG number, All gene number, and the GO accession number. The gradual red color represents $-\log_{10}$ (*q*-value) of enrichment analysis.

.

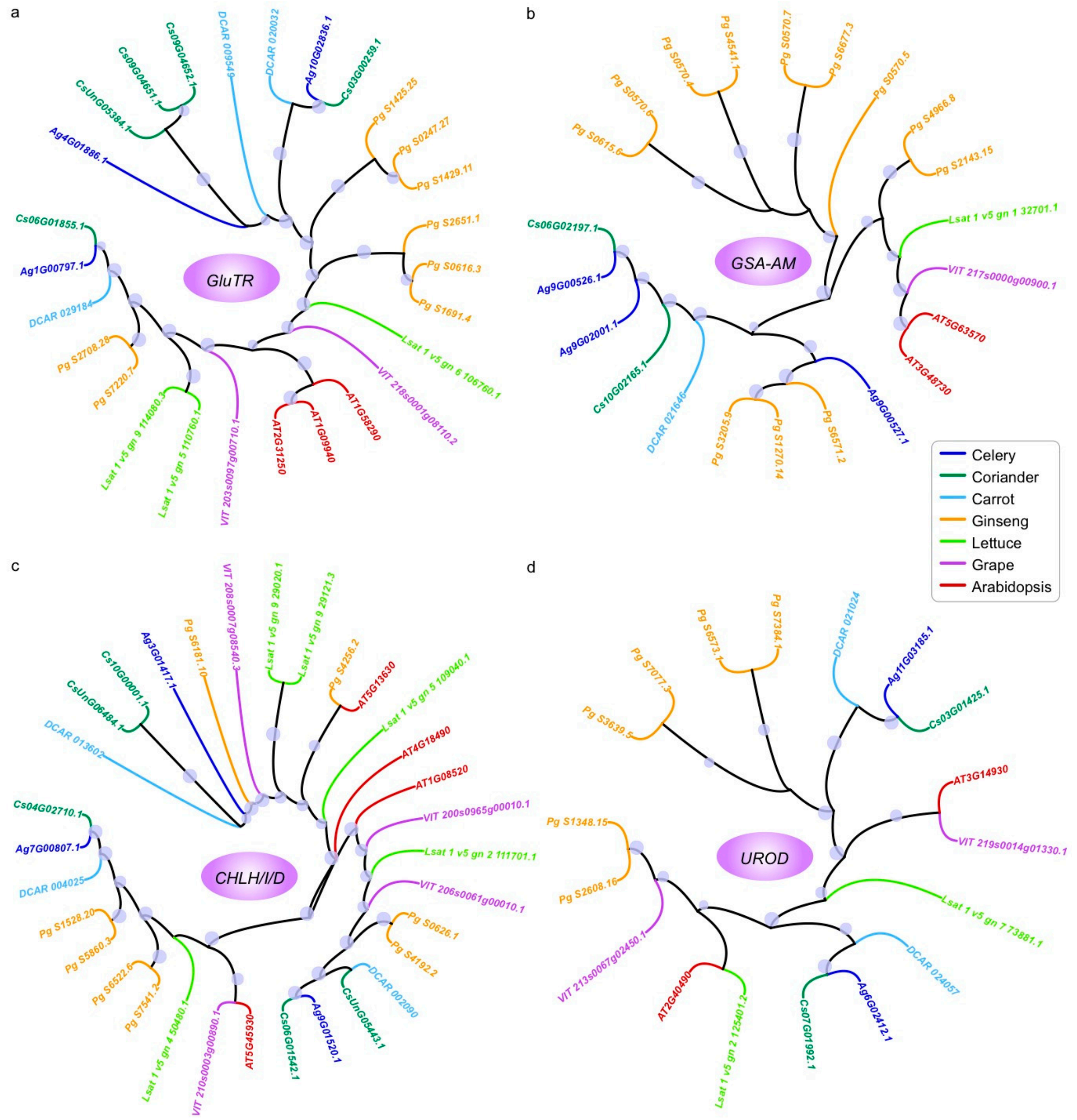

Figure S3. The maximum-likelihood (ML) tree of chlorophyll biosynthesis genes were generated based on the amino acid sequences with 1,000 bootstrap repeats in seven species. The size of the circle on the branch represents the value of bootstrap with ranges from 40 to 100. (a) *GluTR* genes. (b) *GSA-AM* genes. (c) *CHLH/I/D* genes. (d) *UROD* genes.

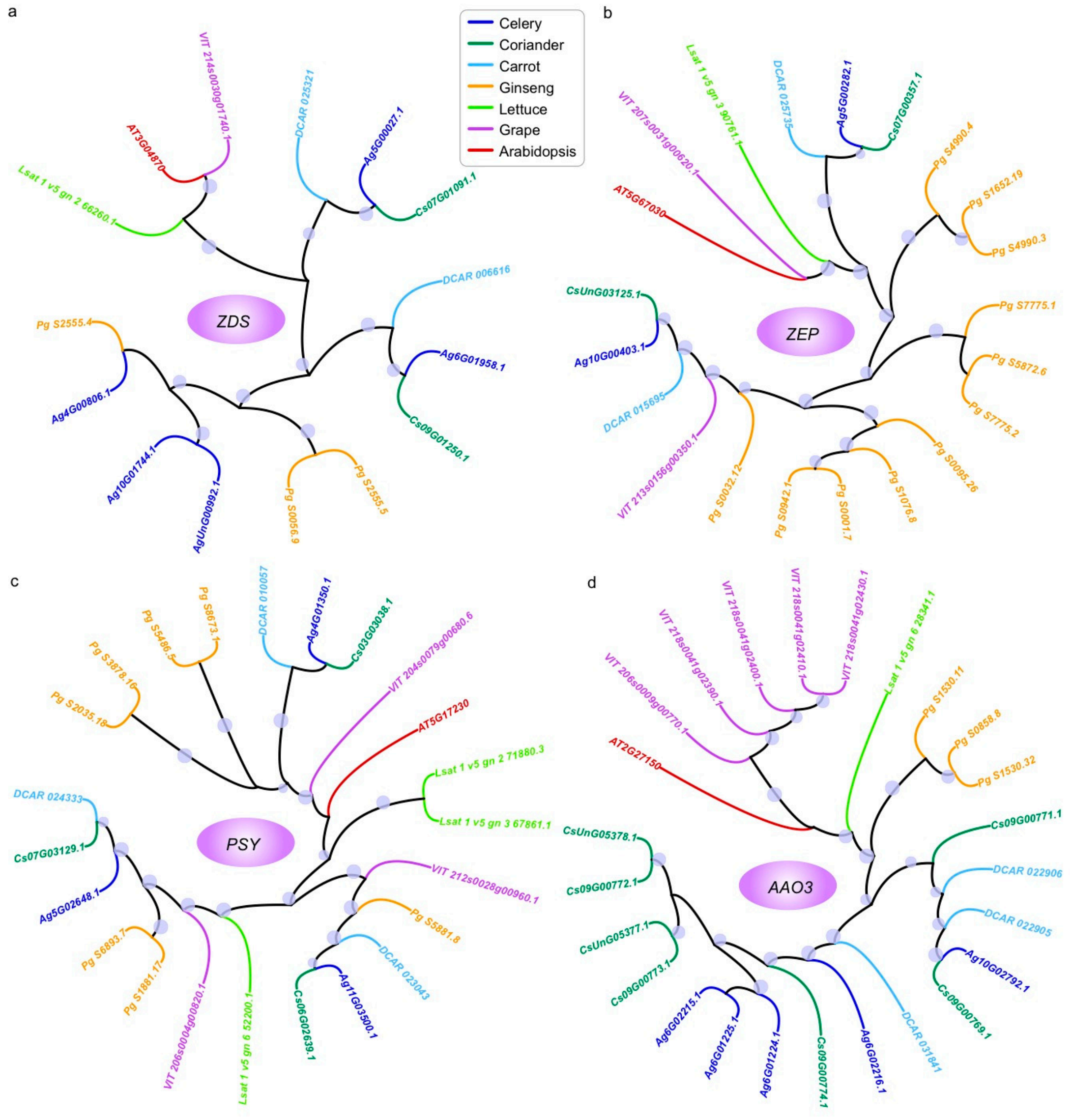

Figure S4. The maximum-likelihood (ML) tree of carotenoids biosynthesis genes were generated based on the amino acid sequences with 1,000 bootstrap repeats in seven species. The size of the circle on the branch represents the value of bootstrap with ranges from 40 to 100. (a) *ZDS* genes. (b) *ZEP* genes. (c) *PSY* genes. (d) *AAO3* genes.
